# Smartphone Architecture for Edge-Centric IoT Analytics

**DOI:** 10.3390/s20030892

**Published:** 2020-02-07

**Authors:** Bockarie Daniel Marah, Zilong Jing, Tinghuai Ma, Raeed Alsabri, Raphael Anaadumba, Abdullah Al-Dhelaan, Mohammed Al-Dhelaan

**Affiliations:** 1 School of Computer & Software, Nanjing University of Information Science & Technology, Nanjing 210-044, China; bockarie.marah@gmail.com (B.D.M.); zljin@nuist.edu.cn (Z.J.); alsabriraeed@gmail.com (R.A.); atiimanaadumba200@gmail.com (R.A.); 2 College of Computer and Information Sciences, King Saud University, Riyadh 11362, Saudi Arabia; dhelaan@ksu.edu.sa (A.A.-D.); mdhelaan@ksu.edu.sa (M.A.-D.)

**Keywords:** edge computing, internet of things (IoT), smartphone, artificial neural networks (ANN), cloud and regression analysis

## Abstract

The current baseline architectures in the field of the Internet of Things (IoT) strongly recommends the use of edge computing in the design of the solution applications instead of the traditional approach which solely uses the cloud/core for analysis and data storage. This research, therefore, focuses on formulating an edge-centric IoT architecture for smartphones which are very popular electronic devices that are capable of executing complex computational tasks at the network edge. A novel smartphone IoT architecture (SMIoT) is introduced that supports data capture and preprocessing, model (i.e., machine learning models) deployment, model evaluation and model updating tasks. Moreover, a novel model evaluation and updating scheme is provided which ensures model validation in real-time. This ensures a sustainable and reliable model at the network edge that automatically adjusts to changes in the IoT data subspace. Finally, the proposed architecture is tested and evaluated using an IoT use case.

## 1. Introduction

The Internet of Things refers to the concept of connecting electrical/electronic devices to the internet. These devices include coffee makers, microwaves, watches, etc. The general idea is to allow these devices to interact with their operating environment by collecting contextual data about their physical surrpondings using sensors. The collected data is then analyzed to derive useful information that can improve their operational efficiency. However, it is clear that the traditional approach to this process is not effective except with the introduction of the edge computing techniques which aim at displacing as many of the analytics as possible towards the edge of the network [1,2]. This study is therefore focused on applying edge computing techniques to process IoT data on smartphones. An edge-centric IoT architecture on smartphones that ensures real-time data capture, processing and model evaluation at the edge of the network is investigated. The processes on the smartphone mentioned above would be carried out while maintaining an efficient communication link with the cloud/core for model creation and updating. The architecture uses the conceptual data collected by the smartphone sensors to build regression models that reflect all the features of the local data subspace. The models would then be deployed on the smartphone to evaluate queries coming from the same data subspace at the edge of the network. This is done to actualize the edge computing solutions on smartphones at the network edge while improving the overall performance of the IoT solution.

Smartphones are amongst the most used devices in the world as they are equipped with many applications that have made them ubiquitous in our daily lives. Moreover, like traditional IoT systems, smartphones have multiple sensors for collecting conceptual data, very complex operating systems that are capable of running complex algorithms and multiple communication options including Bluetooth, WI-FI, infrared, cellular radio, etc. In addition to these components, smartphones can also be used to deploy IoT applications that allow users to interact with the solutions that the IoT provides. All these make smartphones most suitable for deploying IoT solutions ranging from lightweight to very complex ones.

However, IoT analysis is based on datasets from sensors which are analyzed to obtain useful insights that can be used to solve real-world problems. The authors in [3,4,5] introduced some novel methods for data collection in IoT systems taking privacy, energy and data integrity into account. Nevertheless, the traditional approach to IoT data analysis requires sensor data to be sent to the cloud for further processing. This research, however, is focused on the application of IoT on smartphones which are small portable devices that can in no way measure up with the resources on the cloud. This is why it is much more efficient to allow the cloud to handle model building and data storage. This concept was introduced in [6,7] where techniques such as joint optimization and blockchain were used to offload computational tasks to edge computing devices (ECDs) in an IoT system. However, in this architecture, we would rather consider offloading computational tasks to the cloud to supplement the resource-constrained edge device (smartphone) at the network periphery. The research challenge, therefore, is to develop an effective means for creating and updating models in the smartphone IoT system that still meets the characteristics of baseline edge analytic solutions.

Unlike traditional IoT systems, the smartphone has all the components on the same device which means data capture, machine learning modelling and application deployment can all be done on the smartphone at the edge. This raises the importance of using this device for creating IoT applications and hence the need for this architecture. Nevertheless, the proposed architecture requires the cloud for model creation and update but should also maintain the characteristics of the baseline edge analytic systems. To do this, default to fitted model styled architecture is adopted. This simply means that the IoT application would be initiated with a default model that is gradually being updated by retraining with real-time data captured by the smartphone sensors. With this, both the continuously updating model and the updating strategy can be deployed along with the IoT application on the same smartphone. This would ensure a system that performs real-time data evaluation and other data interactive analytic tasks much more efficiently at the edge of the network whilst still maintaining all the characteristics of an edge analytics solution.

However, there are different categories of users for a particular IoT application and hence, any application should reflect characteristics of the different categories in the application subspace. Moreover, one of the main problems of sending all sensing data to the cloud for creating general models over all data is that the generated model would not reflect the unique characteristics of these different categories. This would result in the models making biased decisions for the different categories or even make poor inferences thus degrading the overall performance of the application. To avoid this in the proposed architecture, the application subspace is divided into all its categories and a decision-making model created for each. New users are then registered into these categories and their smartphones initialized with the model artefacts and parameters of the associated category. This scheme would be explored in detail in later sections.

### Related Work

Smartphones have been used extensively to deploy IoT applications that provide solutions to numerous real-world problems [8,9,10]. These applications make use of the sensors on the mobile phone to monitor and collect data from their immediate environment and present the results of the analysis as an application on the smartphone. One of the popular use cases is human activity/posture detection in which the researchers use the positional sensors on the smartphone to detect the posture/activity (e.g., working, standing, sitting etc.) of the user at a particular point in time. The authors in [11,12] proposed a novel method for human activity recognition that uses a filtering algorithm to pre-process the sensor data before activity classification with multi-strategy feature fusion. Moreover, data collection was done by using the gravity, gyro and altitude sensors on an Android smartphone. Other studies [13,14,15,16,17] proposed alternative methods for classifying user activity/posture using either a single or multiple positional sensors on the smartphones to capture data. However, each of the studies used different machine learning algorithms for the classification achieving very high accuracies with little discrepancies between there results. The resulting classification models were deployed on smartphone applications for performing the classification in real-time. Even though these were not full-scale IoT solutions, they bring out the potential for using smartphones in any IoT application either for data collection, data processing or even application deployment. The authors in [13,14] used the smartphone sensors to stream data to the cloud and display them on the screen of the device. The data collected were then processed and placed in public domains for use by other researchers in related fields. 

Nevertheless, the use of smartphones goes way beyond data collection and application deployment as they are equipped with all the components necessary for building full-scale IoT systems. The authors in [11,18] acknowledged the connectivity options available on smartphones and suggests there use as a gateway device in an IoT system. Also, the study reported in [19] used old smartphones as a gateway in an IoT application making use of the various connection options (Bluetooth, cellular, Wi-Fi, etc.) on the device to connect low power BLE sensors to the internet. A mobile application was used for providing an interface to the hardware sensors for streaming data directly to the cloud. These architectures realize the use of smartphones in an IoT system but still does not utilize its computing capacity for performing data processing tasks. Moreover, streaming data directly to the cloud invites new problems including increase communication overhead, high latencies, privacy and even data inconsistencies in the case of intermittent links to the cloud. These problems led to the incorporation of edge computing into IoT architectures which aims at bringing as much of the processing to the network periphery as possible. The authors in [20,21] proposed a privacy scheme called BalancePIC which aims at preserving a balance between privacy, data integrity and computational cost in edge-assisted IoT devices. In addition to this, researchers in [2] introduced a methodology for parametric regression analysis at the network edge where they defined algorithms for building regression models at the edge and selectively forwarding the parameters and other statistics to the gateway and/or cloud to support decision making during query evaluation. Such methods provide solutions to the problems associated with the traditional IoT architectures that directly forwards the contextual data to the cloud.

However, in this research, the goal is to implement this architecture on smartphones so as to make the most of its resources in an IoT system. Creating machine learning models is computationally intensive, even for smartphones, and hence the need to migrate this task to the core/cloud. The created models can then be deployed on smartphones, which continuously evaluates the performance of the deployed model and performs data collection and pre-processing tasks while evaluating queries at the user end. It sounds like a lot but clearly defines the extent to which the smartphone could be used for such tasks. This is evident from the partial use cases mentioned above that only use the smartphones to capture data and deploy models/applications or simply as a gateway that relays captured data to the cloud and displays it on its screen. 

Reference [22] proposed a new architecture for IoT systems that incorporates the use of edge computing. It detailed all the components involved and their functions in the system emphasizing the need for edge analysis in the mix. However, model evaluation and updating schemes were not included in the proposed system as suggested in [2]. This is a very important aspect of an IoT system as multidimensional conceptual data suffers from data bursty and statistical transiency. This means sensed data values expires within a short time while statistical dependencies disappear between them. In this research, however, the concept of sustainable machine learning is utilized to deploy a default model on the smartphone that slowly evolves and adapts itself to the IoT application environment. This concept was partly used in [23] to develop a novel method for meteorological data prediction (Sliding Window-Based Support Vector Regression (SW-SVR)). The concept of selective data forwarding was used as proposed in [24], to design a scheme for forwarding data to the cloud with the aim of reducing the communication overhead and data forwarding cost.

In the remainder of this study the data collection and preprocessing schemes used in the architecture will be defined in Section 2. Then, the model creation scheme is explained in Section 3 The structure of the smartphone IoT architecture (SMIoT) is described in detail in Section 4 and the model evaluation and updating methods discussed in Section 5. In Section 6 an experiment is conducted with a use case on which we would implement all the schemes in the proposed architecture and evaluate its performance. Finally, conclusions and future trends for this study are presented in Section 7.

## 2. Data Collection and Preprocessing

**Figure 1:**
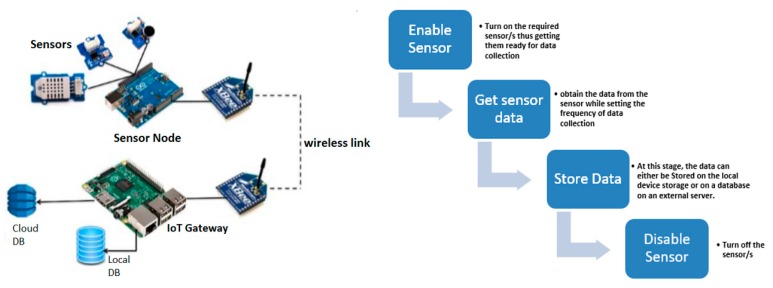
Traditional IoT components for deploying IoT solutions (**Left**). Data collection process by the SMIoT architecture (**Right**).

Traditional IoT systems consist of a sensor network and a gateway either linked by a wired or wireless connection. The gateway is connected to the cloud via the internet as mentioned in [18,25]. The sensor network consists of the sensors connected to a sensor board which is most times implemented using a hardware setup (e.g., Arduino board). A sensor network setup would consist of many different sensors connected to a single hardware board which then aggregates the data and sends it to the gateway via a low power wireless link. Moreover, the wireless links are also implemented using separate hardware devices (e.g., XBee/BT modules) with a low energy consumption rate and hence a low bandwidth. They channel the aggregated data from the sensor boards to the gateways. Figure 1 (Left) shows a typical wireless sensor network (WSN).

The gateways, however, are high powered hardware devices with high computing power and storage space. In most IoT systems, a Raspberry pi is used for this task as it can run an operating system and has a larger memory. The gateways can collect and process data before sending to the cloud for more computationally intensive tasks. 

However, the smartphone is an all in one device that host the sensors, act as the sensor board and the gateway all at once. Smartphones have a well-developed operating system (Android, IOS) with many embedded sensors and multiple connectivity options (Wi-Fi, BT etc.) which makes them suitable for building IoT applications. Moreover, the smartphone is one of the most used electronic devices as it has found its way through many different channels to make it ubiquitous in people’s daily lives. This makes them useful for deploying IoT applications that provide solutions to complex daily problems. 

Nevertheless, for data collection, it is only a matter of having the right tools that can interface with the various IoT components on the device. There are many tools out there but one of the most efficient, is the Ionic Framework that uses Angular (JavaScript framework), React or Vue to write programming constructs for creating mobile, web and desktop applications. According to the android native documentation, the Android OS can host many sensors for measuring different parameters including but not limited to: acceleration, relative humidity, ambient temperature, light intensity, gravity, pressure and proximity. 

Figure 1 (Right) shows a flow chart for data collection in the proposed architecture. The first action is to enable the sensor to ensure that it is “ON” and ready for data extraction. The next process is a more technical one which involves defining the window size, period and granularity of the particular window. Each reading of the sensor is then stored for future use. Storage can either be done on a programming construct, the local device (smartphone) or a database on a web server. This data can then be accessed when needed for preprocessing, or any other real-time data exploration. Finally, the sensor/s is/are disabled to save energy since they consume a lot of the smartphone’s energy during their operation. 

Moreover, smartphones are capable of performing complex operations and also, with the right tools, the data preprocessing can be easily done. However, for the SMIoT, the data from the sensors need to be processed in the following ways before anything else. These processing tasks should be done both on the smartphone after data capture and on the cloud during machine learning modelling. The processes include:

- *Univariate analysis*: analyzing the nature and type of the captured sensor data using a data model
- *Missing data treatment*: detecting missing data and implementing strategic solutions to deal with them (the solution depends on the application use case)
- *Outlier detection and treatment*: detecting and treating captured sensor data that falls outside the range of the other values for a particular sensor (the action taken to treat outliers also depends on the application use case).

## 3. Model Creation and Deployment

This research focuses on regression analytic problems [2] by learning the relationship between the inputs (x) and their corresponding output (y) in a (d+1) dimensional data space $\left(x,y\right)\;\in R^{\left(d+1\right)}$ using ANN. The ANN model can take in any amount of inputs $x=\left(\left[x_{1},x_{2},\ldots x_{n}\right]\right)$—which can refer to any physical quantity (light, relative humidity etc.) that can be measured by sensors on a smartphone—and produce a predicted output $\left(y^{-}\right)$ (given by y=f(x)) with minimal error after learning the relationship between the inputs and the outputs. This model can then be deployed on a smartphone application to evaluate regression queries represented in the form $y^{-}=f\left(q\right)$ for query points $q\in R^{d}$ and a predicted error of e(q)=y−f(q). For example, given a model with humidity $\left(x_{1}\right)$ and light $\left(x_{2}\right)$ measurements as inputs and ambient temperature as output (y), predict the temperature $\left(y^{-}\right)$ given query points $q=\left(q_{1},q_{2}\right)$ where $q_{1}$ and $q_{2}$ are the humidity and light values for which the prediction is to be made.

This study proposes a default to a fitted model styled architecture [23] which aims at using a pre-collected dataset $\left\{{\left(x,y\right)}_{i}\ldots \right\}\in R^{d+1}$ to train default regression models $y=f_{i}\left(x\right)$ for each category $\left(C_{i}\right)$ in the application subspace. At the end of the default model creation, there would be several saved models given by $M=\left\{y_{c_{1}}\ldots y_{c_{n}}\right\}$ in the core/cloud. Their corresponding references, however, would be saved with their respective model_ids, normalizers and error_margins  in a relational database. Since the research is using ANN to train the regression models, the saved models would store two files; the *model topology* (T) and the *model weight* (W) files. The model creation is handled by a server-side script which has links to all the necessary APIs for creating an ANN model.

To deploy these models, a server-side script would be created to automatically register a new user to a particular category (as defines in the initialization request) and returns a response containing the model reference, normalizers and error margin back to the caller. This default model would continually be evaluated at the deployed environment (the smartphone at the network edge) by using the error_margin in the server response to *score* predictions of real-time data captured by the smartphone sensors. Discrepancies are automatically reported to the core/cloud which would retrain the model with the dataset that produced the discrepancy and update the default model with the new one. This process would continue until the model saturates (i.e., the model would be performing at its best and hence would require no further updates). This process ensures that any change detected in the data subspace are automatically reflected in the deployed model. Moreover, apart from evaluation, the smartphone provides the interface for users to query the model. Query results are evaluated very quickly since the inferencing is done at the network edge using the cached model data on the local storage of the smartphone. Model evaluation and update is explained in detail in Section 5.

## 4. Detailed Structure of the Smartphone IoT Architecture

The goal of this research is to deploy and maintain a decision-making model (the ANN models defined above) on the smartphone to support real-time analysis at the edge of the network. The decision-making model on the smartphone, however, is created and updated at the cloud which has a more sophisticated computing system that runs on reliable power sources. It is therefore required for the architecture to provide methods for data transmission between the smartphone at the edge and the core computer. Nevertheless, it is a prerequisite for the system to meet the requirements of standard edge analytics solutions and hence data transmission should be checked. To satisfy this requirement, the smartphone IoT architecture should utilize the process of selective data forwarding [24] which as the name suggests—allows for data to be transferred only when it is utterly necessary. Moreover, as mentioned in previous sections, this architecture would apply the default to fitted style to implement its edge analytic IoT solution.

Following all these conditions, to implement the SMIoT architecture, a default model, created with pre-gathered data, would be stored on the core computer. Smartphones with the specific IoT application would then be initiated with this default model. The model is then sent via a wireless link and later cached in the local storage of the smartphone device. The default model data contains three main components:

- *Model identifiers (name and id)*: These are used at the edge to identify the model that the smartphone should interact with. It is created during model initialization. The *model id* also refers to the category to which the model belongs and hence, it would be used to service all users in that category.
- *Normalizers*: Normalization values used at the edge to do and undo normalization of the model parameters so as to maintain the characteristics of the original model residing at the core.
- *Evaluators*: Evaluation parameters used at the edge to determine when to request updates from the core.

### 4.1. Core Components

**Figure 2:**
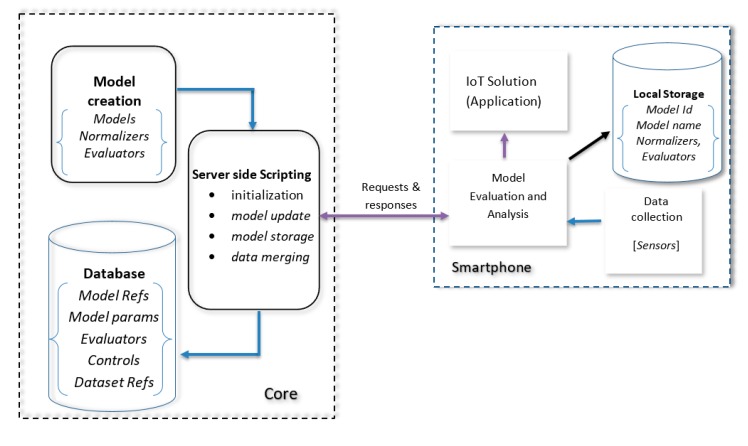
SMIoT components communication links, relationships and processes.

**Figure 3:**
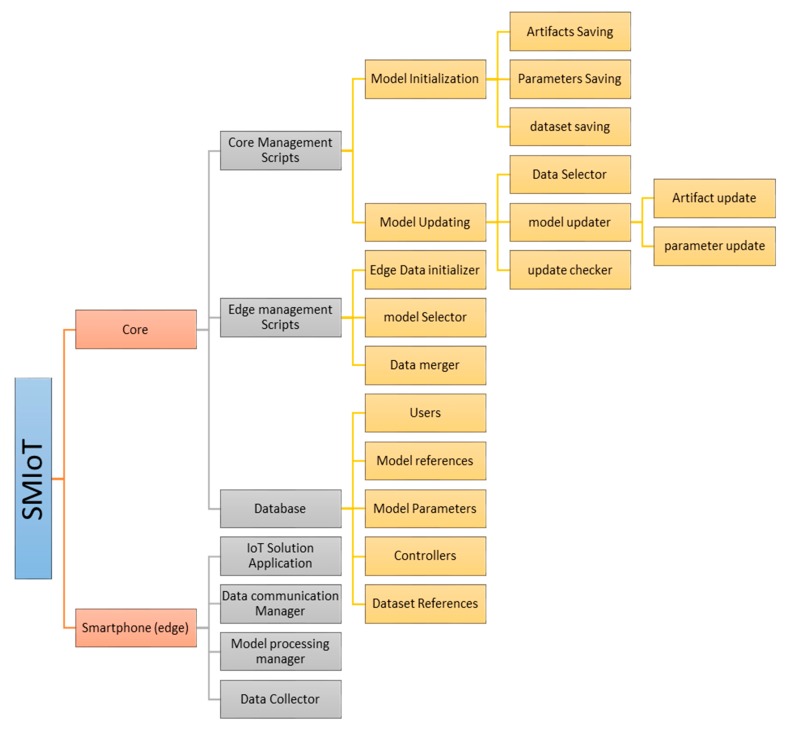
A Detailed structure of the SMIoT architecture showing all components for both core and edge (smartphone) devices.

As can be seen in Figure 2 and Figure 3, the system is initiated at the core where the *model*, along with its *normalizers* and *evaluators* are created and stored in a database. The SMIoT uses server-side scripts to carry out the process defined in both the core and the smartphone at the edge of the network. However, the core is divided into three sections; 

#### 4.1.1. Core Management

The core management section performs tasks relating to the core processes. These include creating and updating the decision-making models for the different categories of the solution application. These two operations are handled by the Model Initializer and the Model updater respectively: 

• *Model Initializer*

The model initialization script uses the default dataset/s to create the default model/s for the respective categories in the solution application. The resulting model/s along with their parameters and dataset are saved at the core using the *artefact*, *parameter* and *dataset* saving scripts, respectively. However, because of the complex structure of the models, only their references are stored in the database while they are themselves stored as files in a separate location on the core computer. The datasets for each model created by the initializer, are organized and stored in another separate location and the references to their location and models stored in the database. The model parameters, however, are stored as literals in the database.

• *Model Updater*

This script is responsible for retraining the locally deployed decision-making models when the need arises. The resulting model and variables after the retraining process would be updated in the database for the particular category and later served to the associated users. Moreover, the model updater manages the *data selection* and *updating checking* scripts. The *update checker* goes through the database to check for models that need to be retrained. The *data selector*, however, is responsible for fetching the dataset of the model that needs retraining so that it would be updated by the *model updating* script. Moreover, the model and its parameters are updated by the *artefact* and *parameter* updating scripts respectively.

#### 4.1.2. Edge Management

This section handles communication between the core and the smartphones. They are responsible for servicing initialization and update requests at the core. These two processes are handled by the *edge data initializer* and *data merger*. During initialization, the *edge data initializer* would process the initialization requests from the smartphones at the network edge by registering them to the appropriate categories in the system. The appropriate data that would support the SMIoT processes at the network edge, is then sent as a response to the smartphone making the request. The *data merger,* on the other hand, handles update requests from smartphones. However, they do not perform the model updates themselves. They simply append the update data coming from the smartphone (registered to a particular category in the system), to the dataset associated with the model of the related category. This data is then used to retrain the model during the model update in the core management section. The *model selection* script assists the *data merger* in the selection of the appropriate model during initialization and data merging.

#### 4.1.3. Database

For reduced complexities and more coordinated access to the model and the other parameters, the core uses relational database management systems (RDBMS) to store related information. The SMIoT database contains the following tables:

- *Users*: contains all the users of the current system and their corresponding model categories and status
- *Model References:* pointers to the locations of the different models in the current system
- *Model Parameters*: the normalizers, margins and error measure of each of the models in the system
- *Controls*: this holds the current status of the models indicating whether or not it is in the updating mode at a particular point in time. This is to prevent interruptions during model updates.
- *Dataset references*: this table holds the pointers to the datasets for each of the models in the system. It is most useful during model updating and data merging.

### 4.2. Edge Components

The solution application is deployed at the edge of the network. The two main operations carried out at the edge of the network includes; query evaluation and model evaluation. However, this section is made up of four sub-components. These include:

• *IoT Solution Application*

This is the IoT application which provides the solution for the users in the different categories. It has a well-defined interface for accepting queries relating to the problem addressed in the IoT use case. The submitted queries are evaluated and the results returned to the caller via the interface.

• *Data Communication Management*

This section handles the communication between the core and the smartphone at the network periphery. It manages the data transaction between the core and the smartphone during initialization and model updating.

• *Model Processing Management*

As mentioned earlier, the smartphone supports all the components of an IoT system on the same device. Therefore, the application front would use the information cached in the *local storage* to perform its normal operation while the evaluation of these variables is performed at the backend of the same device. This section is responsible for evaluating the locally deployed model using the novel evaluation algorithm defined in Section 5.

• *Data Collector*

This section handles the collection of the conceptual data in the application subspace using the sensors on the smartphone (Section 2). The collected data is used in the model processing management section to evaluate the decision-making model deployed on the smartphone (Section 5). This ensures that the IoT application running on the smartphone always have the optimal model and variables necessary for its operations.

## 5. Model Evaluation and Update

Model evaluation is most critical in the SMIoT architecture as it allows the entire system to meet the baseline edge analytic standards. Thus, to materialize this concept, the decision making IoT solution is deployed on the application front while carrying out an evaluation at the backend of the same device using its sensors. 

Conventionally, in IoT systems, the sensors gather data that is used for creating decision-making models for the IoT solution applications. The application then uses these decision-making models to resolve queries emanating from the application domain [2,25]. However, deploying the model alone is not enough for the provision of the IoT solution. This is, because, dependencies between model features change over time and the model would not be able to reflect those changes without a predefined action. To resolve this, the model needs to be evaluated and updated in real-time so that a change in its features would be constantly reflected in deployment. This would make the IoT application adaptive and reliable. As mentioned earlier, smartphones are capable of hosting both the sensor/s and the IoT solution application on the same device. Therefore evaluation and update can proceed in real-time at the edge of the network. The core would only be solicited when there is a need for an update.

### 5.1. Sustainable Machine Learning Models

To materialize real-time model evaluation and updating in a *SMIoT* system, the concept of sustainable machine learning is adopted [23]. A machine learning model is sustainable if it can reflect changes in the data subspace when the changes actually occur. To utilize this concept in the *SMIoT* architecture, evaluation mechanisms are added to the application which conducts the model assessment in real-time and raises flags when any change happens. This is done by deploying assessment matrices during model training at the core. The resulting matrices are sent along with the model artefacts to the smartphone during initialization of the application. These assessments matrices are then used by the smartphone at the edge to define thresholds that will detect changes in the data subspace and raise flags when necessary. When the changes finally occur, the contextual data responsible for the change is shipped to the core for the model update. 

Moreover, to evaluate the regression model which was designed in Section 3, the confidence intervals would be used as it is one of the most effective matrices for assessing predictions for regression models. This matric would be used to create a margin of error which in turn would be used for defining the range of accepted values for all predictions of the deployed model at the edge of the network.

**Theorem 1** **(confidence intervals).** *Given a true value (y), make a prediction $\left(y^{-}\right)$ from the given input that is within the 95% confidence interval range of the true value (y):*

(1) $$confidence\;interval\;\left(CI\right)=y^{-}{}^{*}\pm t_{\alpha /2}Sy^{-}{}^{*}\;$$

*where:*

- *$t_{\alpha /2}$ is the t-score for the 95% CI on a student t-distribution plot of the sample data*
- $Sy^{-}{}^{*}$ is the standard deviation for the particular output $y^{-}{}^{*}$
  (2) $$S_{y^{-}{}^{*}}=S\sqrt{\frac{1}{n}+\frac{{\left(x^{*}-x^{-}\right)}^{2}}{\sum {\left(x_{i}-x^{-}\right)}^{2}}}$$

*where:*

- *S is the standard error of the model (mse),*
- *n is the length of the model dataset used for training and / retraining*
- x* is the input that produces a particular output y
- *x^−^ is the mean of the dataset*

However, the estimated variance of $y^{-}$ is at its minimum at the mean of the independent variable $i.e.,\;x^{*}-x^{-}=0\;when\;x^{*}=x^{-}$:

(3) $$therefore\;S_{y^{-}{}^{*}}=\;S\sqrt{\frac{1}{n}+\frac{{\left(x^{*}-x^{-}\right)}^{2}}{\sum {\left(x_{i}-x^{-}\right)}^{2}}}=\;S\sqrt{\frac{1}{n}+\frac{0}{\sum {\left(x_{i}-x^{-}\right)}^{2}\;}}=S\sqrt{\frac{1}{n}}$$

Therefore, it can be deduced that the most precise estimate of the mean value of y is when $x^{*}=x^{-}$. The confidence interval of the mean of the sample data would be used to set the error threshold of the predictions of the model at the edge of the network.

### 5.2. Evaluation at the Network Edge (Smartphone)

Evaluation on the smartphone is implemented by a simple function that takes as input the confidence interval (CI) of the mean (deduced at the core after model training) and a window of data collected by the sensors on the smartphone. The overall window data contains the following fields:

- *Input values* (x): it is measured by the sensors and it can be any number of inputs (see Section 3).
- *True outputs* (y): it represents the value that the inputs map to. It is also measured by the sensors.
- *Predicted outputs*$\;\left(y^{-}\right)$: these are the model’s guesses at the true output values. They are generated by the deployed model.
- *Error* (ε): this is the difference between the true and predicted outputs.
- *Status* (φ): this is the prediction assessment calculated by the smartphone using the CI generated in Equation (1):
  (4) $$\;evaluatio\;input\;data\left(\beta_{window}\right)=\left\{x,\;y,\;y^{-}\;,\varepsilon ,\;\varphi \right\}$$

The error is given by the absolute difference between the true value and the predicted value

(5) $$error\left(\varepsilon \right)=\;\left|y-y^{-}\right|$$

The prediction status assigns a 0 or a 1 value to a record in the window depending on whether or not the prediction is within the 95%CI of the mean of the sample data.

The evaluation function would perform the assessment at the end of each window which has a fixed size (N) and an overlap (λ) expressed in percentage. The size of the window is fixed in order to reduce the effect of data bursty and statistical transiency of the conceptual data as discussed in section I. With a fixed-sized sliding window, the local decision-making model would always be evaluated with fresh new data which could be sent to the core during an update request. However, N and λ are manually set.

The assessment is made by taking the count of both status 0 and 1 and checking for the one with the highest value. This sets the update request status (γ) at the edge of the network. The process is shown in the Algorithm 1 below.
**Algorithm 1. Model evaluation algorithm (on the smartphone at the network edge)**
**Input**: $\beta_{window}$ (equation 4)
**Output**: update request status (γ)1.**Initialize** good Performance Count & bad Performance Count ← 02.**For each**φ in $\beta_{window}$:3. **If**φ = 0:4.  Increase good performance count by 15. **Else:**6.  Increase bad performance count by 17. **End if**8.**End for**9.**get** total good and bad performance count from 1.10.**If** total bad performance > total good performance:11. γ = true12. Send update data = $\left[{\left\{x,y\right\}}_{i}\ldots {\left\{x,\;y\right\}}_{N}\right]$ of $\beta_{window}$ to the core for model $C_{i}$
13. clear $\beta_{window}$
14.**Else:**15. clear $\beta_{window}$
16.**End if**

The update request contains the input (x) and true output (y) values in the evaluation_input_data(β) window object of size N and the *model id* of the *model* $\left(C_{i}\right)$ for which the update is requested. The *model id* is taken from the local storage on the smartphone which was obtained during the *initialization* process.

### 5.3. Evaluation at the Core

The core/cloud receives the update request data from smartphones of different categories/edge locations and performs the retraining process to update $C_{i}=\left(T,\;W\right)$ and parameters $\left(C_{i\left(p\right)}=\left(normalizers,\;margin\right)\;\right)$. Note that the margin is the 95%CI that was previously obtained in Equation (1). The respective categories are identified by the model_id in the database. As a matter of fact, update request are only sent when the evaluation_input_data window fails the assessment (i.e., has most of its predictions wrong for the real-time data coming from the smartphone sensors). Nevertheless, upon receiving the update_request, the core would automatically append the data to the current model dataset ($C_{i\left(dataset\right)}$) while removing the same amount of records from the same dataset. This technique is applied to keep the length of the dataset constant ($max_{dataLength}$) so as to keep the model dataset with recent data from the associated application subspace. With this approach, the SMIoT architecture would be able to adapt itself to a change in the application subspace whenever they occur. The model update proceeds using the following Algorithm 2.
**Algorithm 2. Model Updating Algorithm at the core**
**Input:** update request data (U) (from **Algorithm 1**)
**Output:** updated $C_{i}$ and $C_{i\left(p\right)}$
1.get $UpdateData\;length\;\left(L_{update}\right)$ from U2.**Fetch**$C_{i\left(dataset\right)}$ using database reference for the model id3.**If**$L_{update}$ < $max_{dataLength}$:4. append U to $C_{i\left(dataset\right)}$
5**Else:**6. discard oldest records from $C_{i\left(dataset\right)}$
7. append U to $C_{i\left(dataset\right)}$
8.**End if**9.retrain $C_{i}$ using appended dataset10.Update $C_{i}\;$& $C_{i\left(p\right)}$ in the database

## 6. Experiments and Performance Evaluation 

The SMIoT system defined above effectively uses smartphones to implement IoT solutions at the edge of the network. To demonstrate this, a light intensity predictor was developed for Android smartphones. The application uses the light sensor on Android smartphones to gather light intensity data from a test Location. The data can then be used to create ANN models that can make up to an hour forecast of the light intensity giving a time interval. This is particularly useful in agricultural applications [26] where an application like this is used to automatically and intelligently control the intensity of the LED grow light/s in a greenhouse. The following sections explain the experimental process and evaluation of matrices.

### 6.1. Datasets

**Figure 4:**
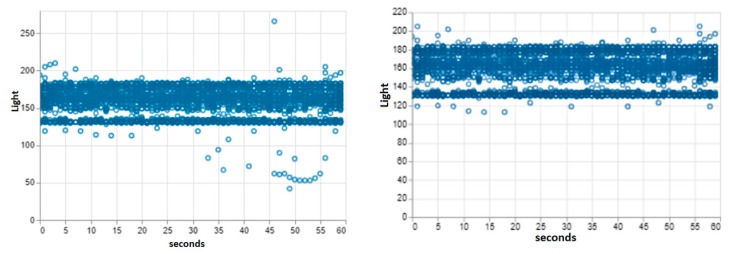
Scatterplot of the default experimental dataset before (left) and after (right) data pre-processing.

The dataset used is light intensity data collected using an Android Smartphone (OPPO A83t Android V7.1.1) in the university lab. This location was, therefore, our test location and hence the default machine learning model created represent the light intensity status of this location. The dataset contains the light intensity values (in Lux) and timestamp (hours, minutes, and seconds) for a time period of 8 hours and granularity of 1 second. The total data collected to train the model was 3066 instances. Figure 4 (left) shows the scatter plot of the hourly and per second changes of the raw unprocessed data captured by the smartphone sensor. Notice that the light intensity for this location falls between the range of 49 and 260 (Lux) with most of the values between 145 and 195 (Lux). This is consistent with the fact that the lab (with an area of almost 100 m^2^) is illuminated with fluorescent bulbs plus a small amount of outdoor light entering through the glass windows. 

Furthermore, this dataset was pre-processed as mentioned in Section 2 to remove outliers, missing values and then normalized before it was made ready for training. To remove the outliers, the dataset was scaled and the z-score calculated for each of the light values. The values with their calculated z-scores exceeding a set threshold (3.0), were replaced with the mean of the dataset. Figure 4 (right) shows the scatterplot of the per seconds changes of the processed dataset.

### 6.2. Training

An ANN regression model that takes in three inputs (timestamp (hours, minutes, seconds)) with the sensed light intensity as the target was created. Tensor Flow JavaScript API (tensorflow.js) was used to build the model for the application. Furthermore, three input neurons and two hidden layers each with 20 units and a softmax activation function was used at the input section of the model topology. These inputs map to one output neuron which uses reLU as its activation function. The training dataset was normalized using the min-max function for which we obtain values between 0 and 1. This was done to get all the instances in the same unit. The training was done using a batch size of 50 for 100 epochs and the mean squared error (mse) as its evaluation matrix. Moreover, the stochastic gradient descent (sdg) was selected as the loss function for the model. At the end of the training, the model artefacts (topology and weight files) were stored in a repository in the core and its reference and evaluation parameters (normalizers, mse and margin) were stored in the database. Note also that the dataset used to train the model was saved as a JSON file in the core while only its reference was saved in the database. 

### 6.3. Test Results

The created model was tested using a reserved dataset that contains records for the 18th hour (6 pm) of the day for the same location (university lab). The novel evaluation algorithm was used to score the performance of the test dataset using the margin to define upper and lower bounds as the model makes its predictions for the test dataset. The evaluation algorithm scores the model 98.248% for this test dataset. 

**Figure 5:**
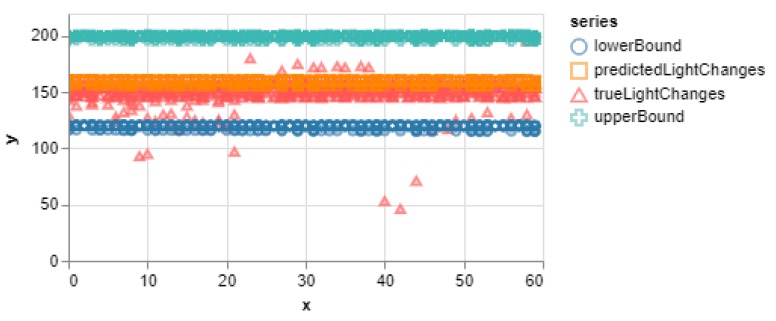
Result of the test dataset for the default model. The x-axis represents the time (sec) while the y-axis is the light intensity in Lux.

Figure 5 shows the evaluation of the test dataset showing clearly the number of predictions that were within the 95% CI band. It can be seen that most of the true values are within the 95% CI band. Hence the overall performance of the model is good as the predicted values are much closer to the true values within the upper and lower margins. Nevertheless, this is the same evaluation algorithm used by the smartphone at the edge of the network to score each window of data so as to determine whether or not to request an update from the core.

#### Testing the Default to Fitted Styled Architecture

**Figure 6:**
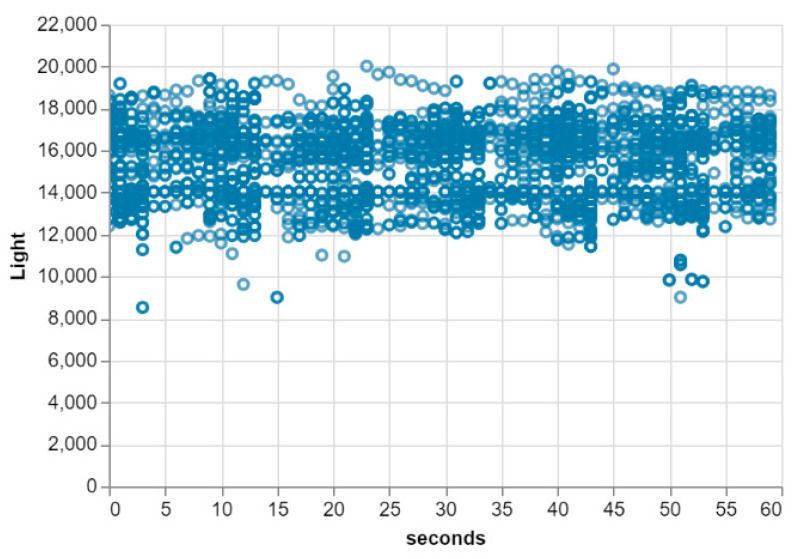
The ANN model dataset after moving the edge device (smartphone) to a new location. Notice the change in the data range from 100–200 to 10,000–20,000.

The SMIoT architecture uses the default to fitted styled architecture to maintain a reliable model at the edge of the network. This ensures that any change in the contextual data is automatically reflected in the deployed model. To test this, an android application was created that forecasts the light intensity for the lab. The app was deployed on two android mobile phones which were automatically registered for the lab model category (test location). One of the smartphones was then taken to the park and was allowed to run for an hour. During this time, the smartphone was evaluating the deployed model in real-time by collecting windows of data (size = 300) and scoring them using the evaluation algorithm. The smartphone would send any window whose score is below a set threshold to the core demanding an update. The core, however, would check for this request according to a set interval and perform the update on the model that was reported. Upon completion, the model artefacts along with its evaluation parameters were updated and redeployed on the registered smartphones. The resulting dataset after several updates is shown in Figure 6. Notice that the range of values changed from 145–195 (Lux) to 8000–21,000 (Lux). This is simply because it was a sunny day in the park where the application was running. This also further confirms that the architecture adapts itself to its operating environment and hence the default to fitted style holds.

**Figure 7:**
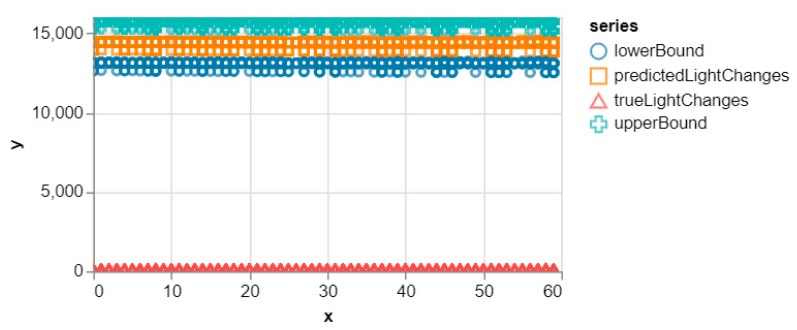
Result of testing the changed model with our default dataset to show the deviations. The x-axis represents the time (sec) while the y-axis represents the light intensity in Lux.

At the end of this period, the test data was run for our test location on the updated model to see how much the default model changed. A performance score of 0% was obtained since none of the samples in the test dataset made it into the 95% CI band of the new dataset as can be seen in Figure 7.

**Figure 8:**
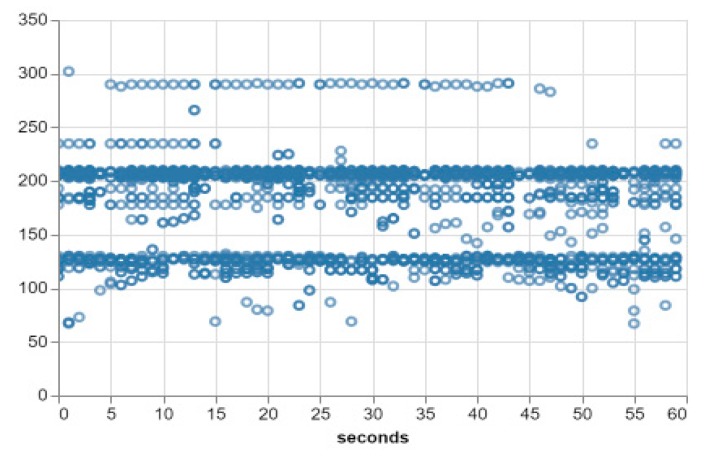
Scatter plot of the final dataset for the tested model category.

**Figure 9:**
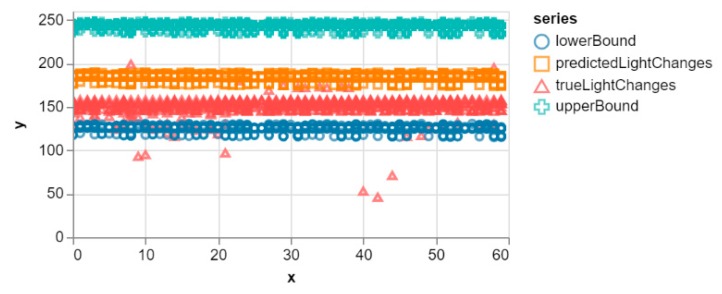
Test result for the default dataset on the adapted model showing the adaptive nature of the proposed architecture. The x-axis represents time (in seconds) while the y-axis represents the light intensity in LUX.

As a final step, two smartphones with the same application were made to operate in the lab (test location). They were both made to use this same model that has just been updated. The two smartphones performed the same process of background window evaluation and scoring as before. However, a function was added to the smartphone application to send the score for each window to the core so we would later have a visual of the process of moving from a period of poor performance to a period of good/satisfactory performance. Figure 8 shows the scatter plot of the final dataset. Notice that the range is within 50–300 (Lux) with the majority of the samples within 145 and 210 (Lux) which is almost the same as the default dataset. The performance score of our test data on the resulting model was 97.3722% as most of the true values fell within the 95% CI band as can be seen in Figure 9.

**Figure 10:**
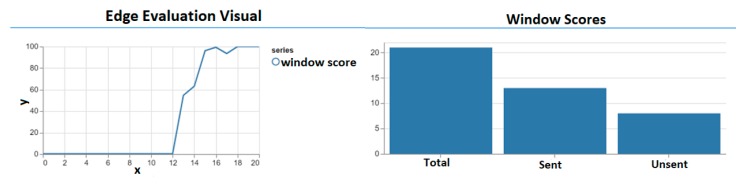
Evaluation of the performance of the model evaluation algorithm on the smartphone (left) (y-axis represents the window score and the x-axis represents the window identity) and the evaluation of the communication overhead by the amount of data sent to the cloud (right).

Moreover, the window scores at the edge were also plotted on a graph and is shown in Figure 10 (Left). Notice the low-performance score of the windows from 0 to 12. From that point on, the scores are very high and therefore do not need updating. As a result of this, the smartphone ceases to send update data. Figure 10 (Right) shows the total number of windows, the amount sent and the amount unsent. It shows that we collected a total of 21 windows of which a total of 11 was sent and 8 were not sent. This shows that our model performs selective data forwarding thus reducing the communication overhead and data forwarding cost which is a typical property of an edge computing solution. However, since the model along with the evaluation parameters is cached on the smartphone (edge) the latency is significantly reduced thus accomplishing another characteristic of an edge computing solution.

## 7. Conclusions and Future Work

This study successfully created a smartphone architecture that performs edge-centric analytics for IoT applications. The architecture defines procedures at the edge for data collection and preprocessing, model evaluation and update and communication protocols to the core that meets the characteristics of an edge analytics solution. Moreover, it also defines processes at the core for model creation and updates as well as data storage for smartphones residing at the edge of the network. The architecture utilizes the process of selective data forwarding at the edge in order to meet the low communication overhead characteristic of an edge computing solution. Moreover, to efficiently outsource model creation to the core, the architecture uses the default to fitted model styled architecture. This ensures that contextual subspace changes are quickly reflected in the deployed model of a particular category (edge location). From experiments performed, the more the smartphones registered to a category, the faster the model adaptation process which is very important since data subspace characteristics and dependencies are always changing in an IoT environment. Also, a novel evaluation and updating algorithm for scoring the performance of a model in real-time was defined. The Light Intensity Prediction use case clearly shows the performance of the proposed architecture as well as all its components and properties.

The experiment performed clearly shows the difference between the SMIoT and the traditional IoT systems which forward all the captured data to the cloud for further analysis. The decision-making models were deployed on the smartphones at the edge of the network to evaluate the user queries. This approach significantly reduces the latency since the queries are evaluated locally as opposed to the traditional systems that use the cloud for query evaluation. Furthermore, to demonstrate the effectiveness of the new model evaluation scheme, the confidence interval, which is one of the most effective statistical matrices for measuring accuracy, was used. The evaluation scheme, (verified by the confidence interval) is used to determine when update requests are to be made in the SMIoT. This ensures that data is only transmitted to the core when it is utterly necessary. This can be seen in Figure 10 (Right) where only 52% of the total data was transmitted to the cloud for the entire period of the experiment. This clearly shows that the SMIoT reduces the communication overhead as opposed to the traditional systems that upload all data (100%) to the cloud for further analysis. Also, the evaluation scheme ensures the sensitivity of the decision-making model to changes in the application subspace. This is illustrated in Figure 5 and Figure 7 where the smartphone quickly detected the change in the environment by using the CI band as a boundary for the evaluated queries.

The experiment was demonstrated on real smartphones and a private server running on the university LAN as the core. Note that, the light intensity prediction is only a use case and not the architecture itself. The smartphone IoT architecture can be used to deploy many more IoT solutions like [27,28,29] and those mentioned in the Introduction section of this paper.

Nevertheless, some sensors are not available on some smartphones and sometimes, even if they are available, they are not as accurate as needed. Moreover, smartphones are often carried in different positions (bags, pockets etc.) and hence it is not too suitable for some applications except if they are dedicated to the application. With all these, it would be more preferable if external sensors are used with the smartphones to collect data while the smartphones perform the analytics at the edge of the network using methods defined in this architecture. Therefore, as a future trend, we would try to link this architecture with external sensors to collect data in a well-coordinated manner.
